# Treatment of Thoracolumbar Fractures Through Different Short Segment Pedicle Screw Fixation Techniques: A Finite Element Analysis

**DOI:** 10.1111/os.12643

**Published:** 2020-03-02

**Authors:** Tie‐nan Wang, Bao‐lin Wu, Rui‐meng Duan, Ya‐shuai Yuan, Ming‐jia Qu, Shuo Zhang, Wei Huang, Tao Liu, Xiao‐bing Yu

**Affiliations:** ^1^ Department of Orthopaedics Affiliated Zhongshan Hospital of Dalian University Dalian China

**Keywords:** Finite element analysis, Pedicle screw system, Thoracolumbar fracture

## Abstract

**Objective:**

To compare the von Mises stresses of the pedicle screw system and the displacement of injured vertebrae using 3‐D finite element analysis, and to evaluate the curative effect of the pedicle screw system.

**Methods:**

Finite element methods were used for biomechanical comparison of four posterior short segment pedicle screw fixation techniques. The different pedicle screw models are traditional trajectory (TT), Universal Spine System (USS), cortical bone trajectory (CBT), and CBT at the cranial level and pedicle screw (PS) at the caudal level (UP‐CBT). The stress distribution of the screws and connecting rods under different working conditions and the displacement of the injured vertebrae were compared and analyzed.

**Results:**

After the pedicle screw system was fixed, the stress under vertical compression was mainly concentrated at the proximal end of the screw, while the stress was mainly concentrated on the connecting rod during flexion, extension, lateral flexion, and rotation. The TT group had the greatest stress during the flexion, extension, and left and right rotation. The UP‐CBT group was most stressed when the left and right sides were flexed; the stress of the USS screw system was less than that of the other three models during flexion, lateral flexion, and rotation. The maximum von Mises stress values of pedicle screws in all exercise states were 556.2, 340.7, 458.1, and 533.4 MPa, respectively. In the USS group, the displacement of the injured vertebra was small in the flexion, and the left and right lateral flexion and the right rotation were higher than in the TT group and the CBT group. The maximum displacements of the injured vertebrae in all motion states were 1.679, 1.604, 1.752, and 1.777 mm, respectively.

**Conclusion:**

Universal Spine System pedicle screws are relatively less stressed under different working conditions, the risk of breakage is small, and the model is relatively stable; CBT screws do not exhibit better mechanical properties than conventional pedicle screws and USS pedicle screws.

## Introduction

According to a recent epidemiological survey, the incidence of spinal fractures is approximately 32.8/100 000^1^, and thoracolumbar fractures account for 90% of spinal fractures. More than 50% of thoracolumbar fractures occur between T_11_–L_2_, which is stress concentration T_11_‐L_2_^(^ 2, 3 ^)^. With the increase in the occurrence of high‐energy trauma injuries, such as from traffic accidents and high‐altitude falls, the incidence of thoracolumbar fractures is increasing. Twenty percent of thoracolumbar fractures are burst fractures, which are usually caused by high‐energy damage, and are due to axial pressure. The buckling rotation force causes the front column and the center column to be unable to support^4^. In 15% to 30% of cases, spinal cord injuries are associated with neurological complications^3^.

The optimal treatment for thoracolumbar fractures remains controversial. Despite potential complications, surgical treatment is very effective, surgical treatment is still very effective when there are severe compression and neurological impairments^5^, ^6^. Surgical treatment can provide an alternative to conservative treatment. Surgical treatment can not only allow early mobilization but also prevent subsequent complications, as well as correct deformities and restore vertebral height^7^, ^8^. Rigid fixation can also reduce nerve compression and improve nerve function^9^. There are many surgical methods for the treatment of thoracolumbar fractures, including anterior and posterior approaches. Direct anterior decompression can provide more direct and complete decompression of the spinal canal, which may lead to better neurological function^10^, ^11^. The anterior approach allows direct decompression of the ventral bone and soft tissue insertion, providing superior spinal canal cleansing^12^, ^13^, but is associated with substantial surgical trauma, long operation times, significant blood loss and many surgical complications, and is a difficult operation. At the same time, anterior surgery can cause ureteral injury, peritoneal injury, and spleen rupture^14^. Many surgeons have recently chosen to use the posterior approach^10^. Posterior surgery is the current standard surgical procedure for thoracolumbar fractures. The implant system is used to make the posterior part of the spine more stable. More frequent applications reflect several advantages of this method, such as less intraoperative blood loss, better postoperative pain relief, and shorter length of hospital stay^10^.

The purpose of treatment for spinal fractures is to restore the original sagittal and coronal planes^15^. Especially in the case of thoracolumbar burst fractures, the method most commonly used to achieve this goal is the pedicle screw system for posterior surgery. In posterior surgery, the pedicle screw system has become the gold standard for spinal internal fixation. If the injury is heavier, the method can be combined with anterior fusion^16^. Although the lumbar pedicle screw fixation system has the advantage of using the biomechanical stability of the pedicle screw, screw loosening still occurs, especially in patients with osteoporosis, leading to failure of correction and nonunion^17^, ^18^.

The pedicle screw was first designed and manufactured by King^19^ in 1949. The pedicle fixation technique was first proposed by Boucher^20^ in 1959 and can be used to fix the three columns. Roy‐Camille *et al*.^21^ (1986) applied pedicle screw implantation technology in the treatment of thoracolumbar fractures, and achieved a good clinical effect. With the rise of biomechanics, research on the pedicle screw system has also progressed, and a variety of pedicle screw internal fixation systems have appeared in the clinic^10^. The commonly used pedicle screw system in clinical practice comprises a short segment of four nails and two rods. A large number of biomechanical studies have been conducted on internal fixation systems using pedicle screws. Among them, Kubosch *et al*.^10^ compared the Universal Spine System (USS) vertebra by mechanical test. The torsional stiffness and failure cycle of the pedicle screw system and the traditional pedicle screw system show that the torsional stiffness and failure cycle of the USS pedicle screw system are superior to those of the traditional pedicle system. Liu *et al*.^22^ simulated the 3‐D finite element model of the lumbar vertebrae of T_12_–L_2_ to simulate the USS pedicle screw fixation model at different depths, and found that the insertion of screws with 60% or more depth can achieve sufficient strength and make the injured vertebrae more stable.

These methods have their own advantages and disadvantages. With the wide application of the pedicle screw system, several years of follow up have revealed that many patients have experienced loose internal fixation, broken nails, kyphosis, and height loss of the anterior edge of the vertebral body. In 2009, Santoni *et al*.^23^ first proposed a cortical bone trajectory (CBT) screw internal fixation technique, which was performed by tilting the pedicle from the inside to the outside, mainly relying on the cortical bone to control the screw. Takata *et al*.^24^ proposed a type of degenerative lumbar spondylolisthesis, in which the upper segment was fixed with CBT screws, and the lower segment was fixed with conventional pedicle screws. The soft tissue in the lower part of the segment was separated, the length of the incision was shortened (the average incision length was only 4 cm), and the surgical injury was alleviated. The lumbar spondylolisthesis was well corrected in 6 patients who underwent the procedure. If this article focuses on the traditional pedicle screw, the USS screw, the CBT screw, and the above four pedicle screw system under the CBT screw for the treatment of thoracolumbar fractures, the biomechanical effect of this experiment will allow finite element analysis.

The objective of the present study is to use the USS pedicle screw system to compare the traditional trajectory (TT), CBT, and CBT at the cranial level and pedicle screw (PS) at the caudal level (UP‐CBT), which are used to treat thoracolumbar fractures, based on the following criteria: (i) stress distribution on the T_12_–L_2_ spine vertebrae and implant; (ii) peak stress of the pedicle screw system; and (iii) smallest axial displacement of the injured vertebrae.

The working hypothesis was that of the four implants, the USS had the best biomechanical performance when used for thoracolumbar fracture fixation.

## Materials and Methods

The software Mimics 20.0 (Materialise, Belgium), 3‐matic 12.0 (Materialise, Belgium), SolidWorks 2014 (Simulia, USA), and Abaqus 6.13 (Simulia, USA) were used. A CT scanner (GE, USA) was used to collect raw data in DICOM format with a scan slice of 0.625 mm.

### *Intact and Fracture Model*

A finite element model of the T_12_–L_2_ spine, which included three vertebrae and two discs, was reconstructed. Geometrical details of T_12_–L_2_ spine vertebrae were obtained from 64 spiral CT images of a 26‐year‐old healthy male (65 kg and 172 cm) with no history of spine injury or osteoporosis or radiographic evidence of degeneration. The CT images were scanned and imported into Mimics 20.0 (Materialise, Belgium). The surface model was then exported into 3‐matic 12.0 (Materialise, Belgium) to generate and enhance the quality of the solid model. Eventually, it was imported to Abaqus 6.13 (Simulia, USA) for meshing and analysis. Each vertebral body consisted of cortical bone and cancellous bone, and each vertebral disc was composed of nucleus pulposus, annulus fibrosus, and endplates. Posterior elements were built separately from the vertebral bodies. Based on Boolean algebra, approximately 60% of the L_1_ segment was resected, we established a 3‐D finite element model of injured vertebrae through the removal of the anterior and middle column of the L_1_ vertebrae.

### *Fixation Models*

**Figure 1:**
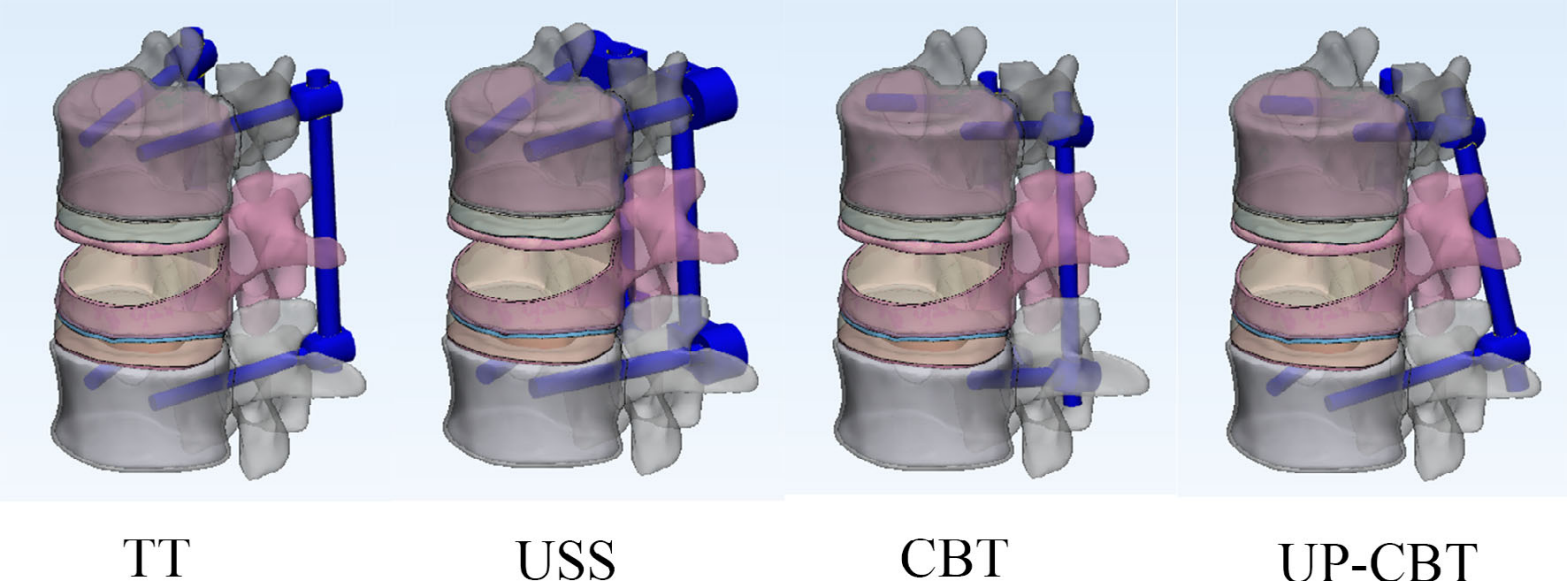
The T_12_–L_2_ finite element models. CBT, cortical bone trajectory; TT, traditional trajectory; USS, Universal Spine System; UP‐CBT, CBT at the cranial level and PS at the caudal level.

Four fixation models were established for unstable thoracolumbar fractures: the traditional trajectory (TT) model, the Universal Spine System (USS) model, the cortical bone trajectory (CBT) model, and the CBT at the cranial level and PS at the caudal level (UP‐CBT) model. The implants and the vertebral structure were precisely engaged. The diameter of the TT screw was 6.5 mm and the total length of the screw was 45 mm. The diameter of the USS screw was 6.2 mm and the total length of the screw was 45 mm. The diameter of the CBT screw was 5.0 mm and the total length of the screw was 35 mm. The fixation models are shown in Fig. 1. The element types, the material properties, and the ligamentary cross‐sectional area are shown in Table 1. Four finite element types were investigated (Fig. 1).

**Table 1:** Material properties of the finite element models

Component	Young's modulus (MPa)	Poisson's ratio	Cross‐section (mm^2^)
Cortical bone and endplate	12,000	0.30	
Cancellous bone	100	0.30	
Annular fiber	450	0.30	
Nucleus pulposus	1	0.49	
Anterior longitudinal ligaments	8	0.30	75.9
Posterior longitudinal ligaments	10	0.30	51.8
Ligamentum flavum	15	0.30	78.7
Intertransverse ligament	10	0.30	36.3
Supraspinous/interspinous ligaments	10	0.30	75.7
Capsular ligament	8	0.30	102
Pedicle screws and rods	110 000	0.30	

A posterior pedicle screw system involving four nails and two rods was used (Table 1).

The entry point and direction of the TT screw are along the vertebral arch, with the root anatomical axis into the nail, parallel to the upper and lower endplates of the vertebral body.

The USS consisted of four Schanz screws and two connecting rods. The Schanz screw can have a sliding support of up to 15° when resetting. The connecting rods of the USS are located directly on both sides of the spinous process and behind the lamina, and spread out to the adjacent vertebrae. Rear support can be improved when resetting, and resetting and fixation can occur without the need to fix the injured vertebra, reducing the dissection and injury of the diseased paravertebral muscle.

In CBT, a relatively new technique for pedicle screw insertion in the lumbar spine, a screw follows a sagittal caudocephalad path and a laterally directed path in the transverse plane to maximally engage the cortical bone from the pedicle to the vertebral body. Given the more medial approach, the new technique is less invasive as it requires a smaller incision and less lateral tissue dissection.

In the UP‐CBT group, the upper segment is fixed with CBT screws, and the lower segment is fixed with conventional pedicle screws. This UP‐CBT procedure requires an incision of 5–6 cm. In terms of incision length, the UP‐CBT screw is less invasive than the traditional trajectory screw.

### *Boundary and Loading Conditions*

The inferior endplate of L_2_ was constrained in all degrees of freedom. A pure moment of 10 Nm combined with a pre‐compressive load of 150 N was applied to the top surface of T_12_. Flexion, extension, left/right lateral bending, and left/right axial rotation were simulated.

### *Rationalities of the Models*

To validate the rationality of the models, including model simplification, material properties, boundary conditions, and loads, a moment of 10 Nm and a compressive load of 150 N were applied at the reference point. These loading conditions are adopted from the biomechanical experiments and published finite element analyses. The range of motion (ROM) among different models was compared. There was little difference between the models. Therefore, the models in the present study are effective for further analyses (Table 2).

**Table 2:** Comparison of range of motion between the current intact model and models from previous studies^25^, ^26^ (°)

Motion	Present	Pflugmachers	Changqing
	study (mean)	study (mean ± SD)	Li's study (mean ± SD)
Flexion	6.1	5.3 ± 1.0	4.6 ± 0.6
Extension	5.3	5.7 ± 1.0	4.5 ± 1.1
Left lateral bending	4.2	4.3 ± 0.6	4.6 ± 0.7
Right lateral bending	4.7	4.3 ± 0.6	4.8 ± 0.5
Left axial rotation	2.6	2.1 ± 0.5	3.2 ± 0.8
Right axial rotation	2.1	2.1 ± 0.5	3.2 ± 0.6

### *Assessment Indexes*

The ROM of T_12_–L_2_, von Mises stress, and the stress nephogram of the pedicle screws and rods of the four fixation finite element models under six loading (flexion, extension, left/right lateral bending, and left/right axial rotation) conditions were analyzed. No statistical analysis was performed in the manuscript because only one subject was modeled.

## Result

### *Comparison of the Force of Pedicle Screw System under Different Working Conditions*

**Figure 2:**
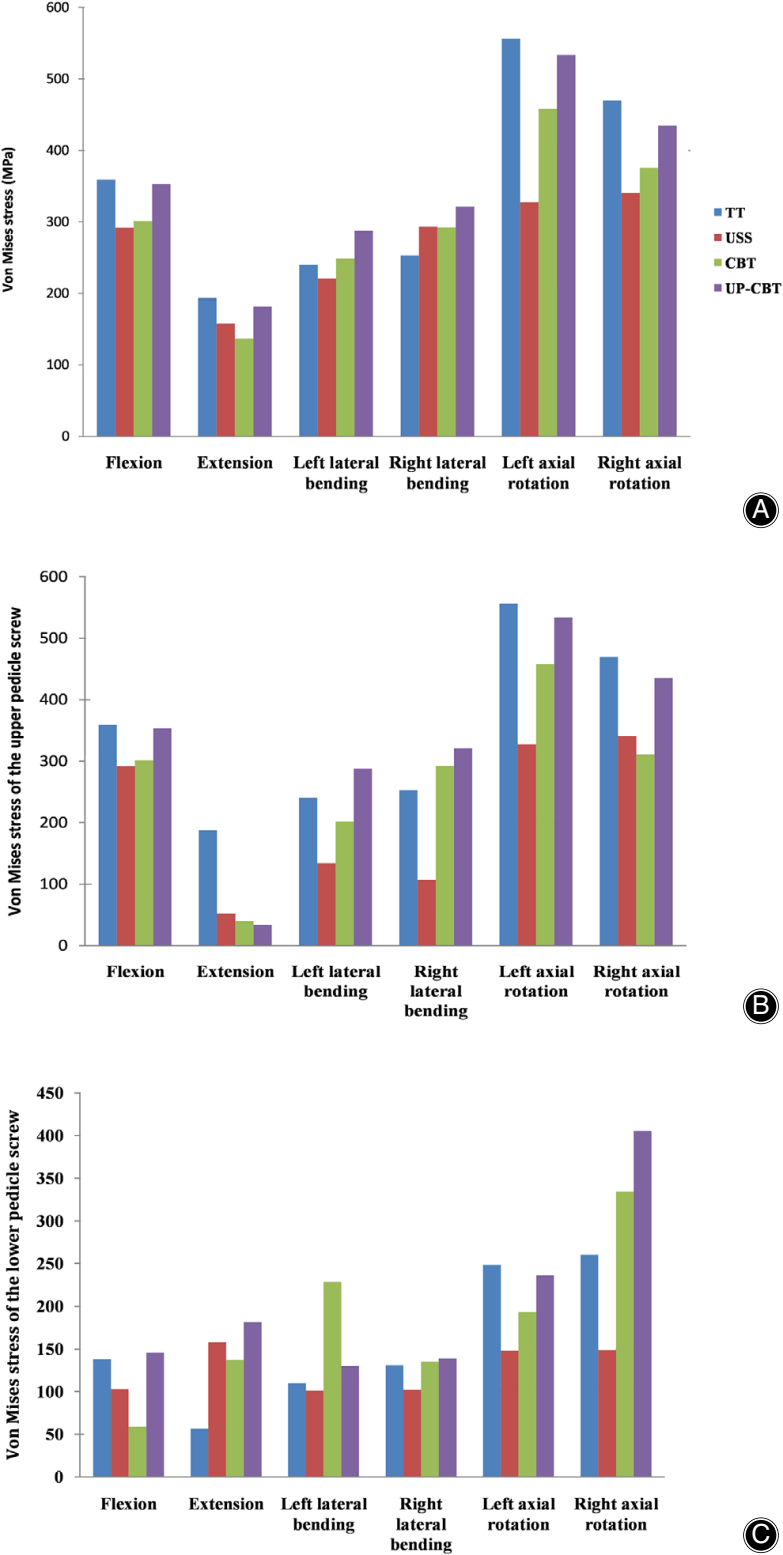
(A) Von Mises stress of the pedicle screws. (B) Von Mises stress of the upper pedicle screws. (C) Von Mises stress of the lower pedicle screws. CBT, cortical bone trajectory; PS, pedicle screw; TT, traditional trajectory; USS, Universal Spine System; UP‐CBT, CBT at the cranial level and PS at the caudal level.

In the four pedicle screw models, the minimum stress was observed in the posterior extension state, and the maximum stress was most significant in the rotating state. The TT group had the greatest stress during the flexion, extension, and left and right rotation. The UP‐CBT group was most stressed when the left and right sides were flexed; the stress of the USS screw system was less than that of the other three models during flexion, lateral flexion, and rotation. In the post‐extension state, the stress of the upper screw is significantly greater than that of the lower screw (Fig. 2).

**Figure 3:**
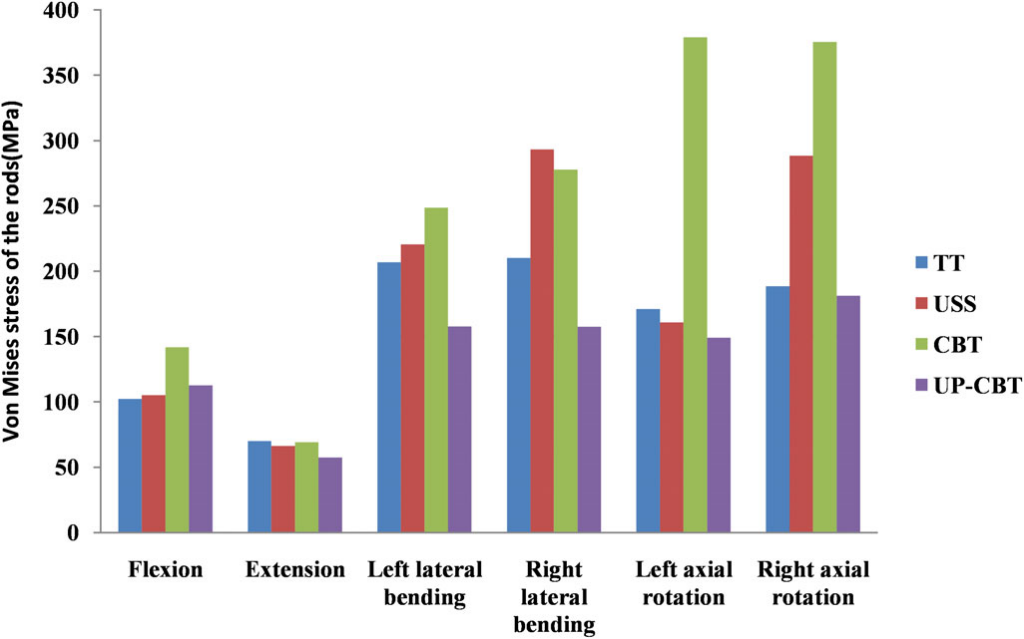
Displacement of injured vertebrae. CBT, cortical bone trajectory; PS, pedicle screw; TT, traditional trajectory; USS, Universal Spine System; UP‐CBT, CBT at the cranial level and PS at the caudal level.

In the TT group, the USS group, the CBT group, and the UP‐CBT group fixed model, the maximum von Mises stress values of pedicle screws in all exercise states were 556.2, 340.7, 458.1, and 533.4 MPa, respectively (Fig. 3).

### *Stress Distribution of Rod under Different Working Conditions*

**Figure 4:**
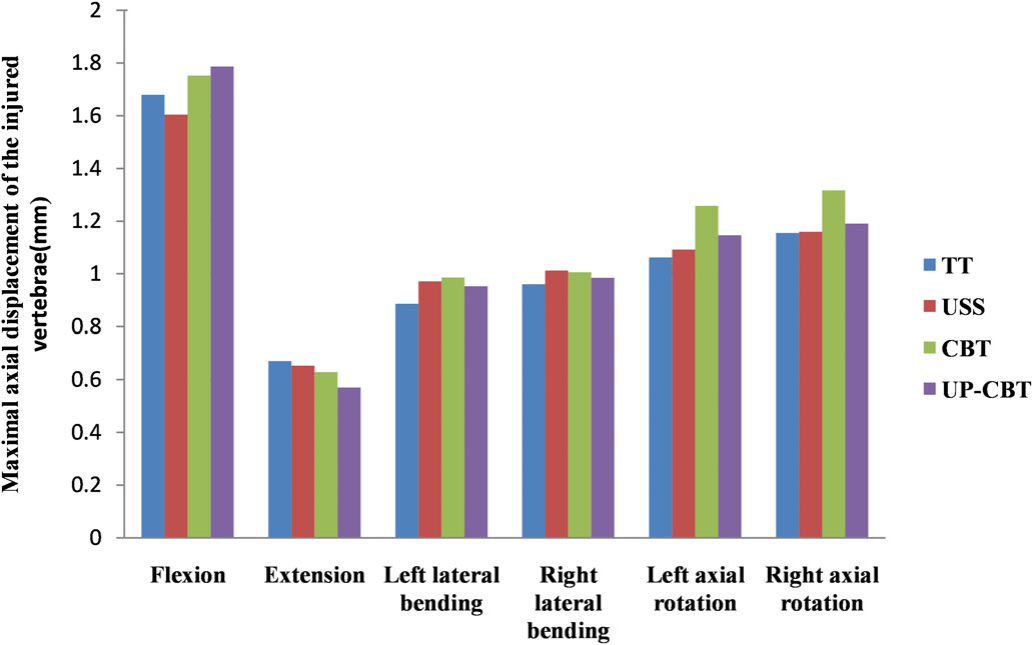
Von Mises stress on the rods. CBT, cortical bone trajectory; PS, pedicle screw; TT, traditional trajectory; USS, Universal Spine System; UP‐CBT, CBT at the cranial level and PS at the caudal level.

During all the motion states, the stress of the rod in the CBT group was significantly greater than that of the other three groups in the flexion and rotation state of the rod. The UP‐CBT group showed relatively little stress on the left side and the left and right rotation. The maximum stress of the rod mostly occurs in the lateral flexion and rotation of the fixed model. In the TT group, the USS group, the CBT group, and the UP‐CBT group fixed model, the maximum von Mises stress values of the rods under all motion conditions were 188.6, 293.3, 379, and 181.2 MPa, respectively (Fig. 4).

### *Displacement of Injured Vertebrae under Different Working Conditions*

**Figure 5:**
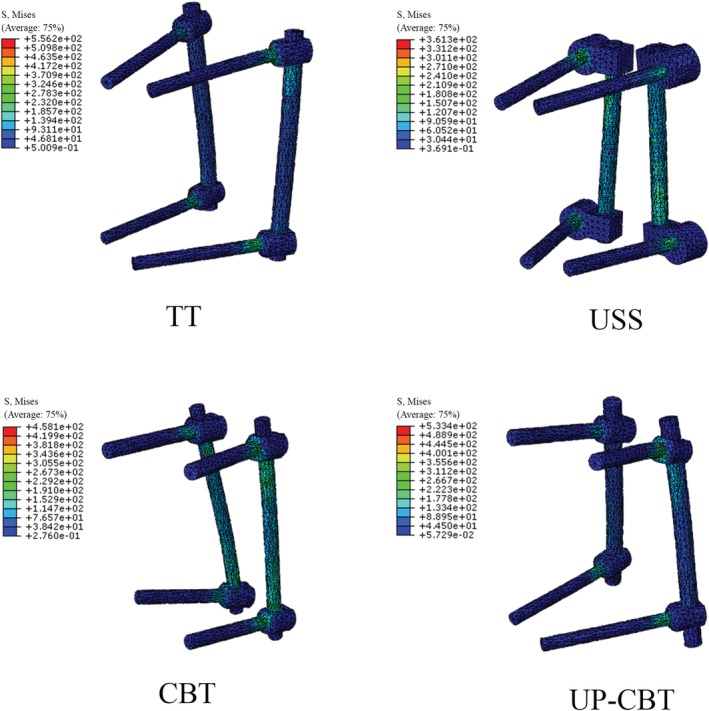
Stress nephogram of the pedicle screws and rods. CBT, cortical bone trajectory; TT, traditional trajectory; USS, Universal Spine System; UP‐CBT, CBT at the cranial level and PS at the caudal level.

In the four models, the displacement of the injured vertebra was the largest with flexion, and the displacement of the injured vertebra was the smallest when the extension was extended. In the USS group, the displacement of the injured vertebra was small in the flexion, and the left and right lateral flexion and the right rotation were higher than for the TT and CBT groups. The displacement of the injured vertebra was the largest when flexing forward and left and right. In the fixed models of the TT group, the USS group, the CBT group, and the UP‐CBT group, the maximum displacements of the injured vertebrae in all motion states were 1.679, 1.604, 1.752, and 1.777 mm, respectively (Fig. 5).

## Discussion

The posterior pedicle screw technique has become the gold standard for spinal internal fixation. Thoracic and lumbar fractures can be treated with a variety of posterior surgical techniques. A posterior pedicle screw system involving four nails and two rods was used. Fixation can improve *reduce* kyphosis, reduce the indirect decompression of the spinal canal, and help to restore nerve function. Although lumbar pedicle screw fixation has the advantage of the biomechanical stability of pedicle screws, screw loosening and rupture still occur, especially in patients with osteoporosis, leading to failure of correction and nonunion. With the development of pedicle screw technology, a variety of pedicle screw systems have emerged in the clinic.

Currently, the USS pedicle screw system is widely used in clinical practice. Some scholars have applied the USS pedicle screw system in clinical practice, and a lot of research has been conducted. Aono *et al*.^25^ used the USS screw system to treat 27 patients with thoracolumbar burst fractures. One year later, the internal fixation was performed. The USS screw system showed good correction of kyphosis, improved nerve function, and fracture reduction. Yang *et al*.^26^ reported that 22 patients with lumbar burst fractures with incomplete neurological injury had improved neurological impairment thanks to the posterior short segment USS pedicle screw system, a well as vertebral height recovery and improved kyphosis. Yang *et al*. also pointed out that the load of the USS pedicle screw system is not transmitted directly from the upper screw to the lower screw but from the upper screw to the connector to the rod to the connector to the lower screw. The load of the traditional pedicle screw is transmitted directly from the upper screw to the lower screw.

Because the rod of the USS is close to the sides of the spinous process and close to the rear of the lamina, it can have a better supporting effect, and can avoid the “parallelogram effect” of the conventional screw, thereby achieving a better resetting and fixing effect. The cortical nail screw internal fixation technique is currently a hot topic. The CBT screw internal fixation technique was first proposed by Santoni *et al*.^23^ in 2009. A screw follows a sagittal caudocephalad path and a laterally directed path. Later, Takata *et al*.^24^ proposed a new procedure; that is, the upper segment is fixed with CBT screws, and the lower segment is fixed with conventional pedicle screws, which can reduce the peeling of soft tissue and reduce the length of the incision. At the same time, many scholars have conducted research on the grip of CBT screws. Santoni *et al*.^23^ compared the axial pull‐out force of CBT screws and traditional pedicle screws through the cadaveric lumbar model and observed the uniaxial pull of CBT screws. The pull‐out test increased the resistance of the pull‐out force by 30% compared to conventional pedicle screws. Matsukawa *et al*.^17^ used a lumbar finite element model and revealed a 26.4% increase in resistance to extraction of CBT screws compared to conventional pedicle screws. However, there is no overall force analysis study of CBT screws. We suspect that if CBT screws are used for the treatment of fractures, the force characteristics would be better than for traditional screws and USS. We use finite element analysis to study the four different pedicle screw and provide a reference for clinical practice.

### *Construction of Finite Element Model of Thoracolumbar Spine*

Cortical and cancellous bones were simulated using 3‐D homogeneous solid elements. The anterior/posterior longitudinal ligament, the transverse ligament, the ligamentum flavum, the interspinous ligament, and the supraspinous ligament were modeled using spring unit elements. We constructed a T_12_–L_2_ finite element model. The lower surface of L_2_ is constrained by all degrees of freedom. A pure torque of 10 Nm was applied in combination with a precompression load of 150 N to the top surface of T_12_. We simulated buckling, stretching, left/rightward bending and left/right axial rotation, consistent with other experimental models. The pedicle screw system simplifies the threading and binds the screw to the vertebral body to avoid failure of the mesh due to the thread and affects the calculation of the model.

### *Constructing an Intervertebral Disc*

Previous researchers have used the Edit Masks function in MIMICS in the construction of intervertebral discs. This function needs to be depicted in the cross‐section of the intervertebral disc according to the shape of the intervertebral disc, and then smoothed. This method is more complicated and there will be errors. In this study, Anatomy Reconstruction, a new function of 3‐matic 12.0 software, was used to form the general shape of the intervertebral disc by constructing the upper and lower vertebral bodies of the upper vertebral body, and then constructing the annulus fibrosus, nucleus pulposus, and end plate.

### *Establishment of the Injured Vertebra Model*

For the finite element study of thoracolumbar fractures, the T_12_–L_2_ segment was used, and the injured vertebra was modeled for L_1_. Partial resection of L1 or partial elastic modulus were conducted and Poisson's ratio for partial vertebral fractures was applied. The finite element method does not precisely model the actual injured vertebral body. To establish a model close to the clinical fracture reduction, a thoracolumbar fracture model of healthy normal people is used to perform the injured vertebrae osteotomy, and the vertebral screw system is used to restore the injured vertebrae. In this study, a V‐shaped osteotomy was performed on 60% of the vertebral bodies on the L_1_ vertebral endplate, and the injured vertebrae model of the anterior and posterior column injury was constructed.

By applying a vertical load of 500 N to the upper surface of the model and a torque of 15 Nm to simulate different motion states such as flexion, extension, lateral flexion, and rotation, it can be observed that in the four pedicle screw models, in the four pedicle screw models, it can be observed that the minimum stress is displayed in the extended state and the maximum stress is displayed in the rotated state. The minimum stress shows the maximum stress in the rotating state. The stress of the screw is the largest in the TT group during flexion, extension, and rotation. The stress in the UP‐CBT group is greatest when lateral flexion occurs. The USS screw system has less stress during forward flexion, lateral flexion and rotation than the other three models. The stress during bending and rotation is smaller than in the other three models. In the extended state, the stress peak of the internal fixator mainly appears in the lower screw, and in the post‐extension state, the stress of the upper screw is significantly greater than that of the lower screw.

For the stress of the rod, the stress of the CBT group was significantly greater than that of the other three groups in the flexion, left flexion, and left and right rotation states. The UP‐CBT group showed relatively little stress on the left side flexion and the left and right rotation. The maximum stress of the rod mostly occurs in the lateral flexion and rotation of the fixed model. Through the force cloud diagram of the pedicle screw, it can be observed that the maximum stress of the pedicle screw system of the four models is concentrated on the proximal rod end of the pedicle screw, which is consistent with the clinically observed screw fracture site. Usually, the junction of the screw and the connecting rod is the most common site of clinical pedicle screw fracture. The fixation of the pedicle screw system stabilizes the spine and promotes healing of the injured vertebra. Early functional exercise is beneficial to patients, but inappropriate or exercise of excessive intensity is very likely to cause pedicle screw fracture. Under the four kinds of exercise state, the maximum stress of the pedicle screw is from large to small: for rotation, flexion, lateral flexion, and extension, respectively. The USS pedicle screw system has the lowest rotation, flexion, and lateral flexion among the four pedicle screw systems, and the risk of nail breaking is minimal. At the same time, bed rest and wearing a brace can reduce the force of the injured vertebrae and limit the range of lumbar motion, as well as reduce the risk of nail breakage.

The displacement of the injured vertebra can reflect the stability of the pedicle screw system. Under the four kinds of motion states, the four pedicle screw system models have the same trend in change of the injured vertebrae displacement. The maximum displacement of the injured vertebra is from large to small. For flexion, rotation, lateral flexion, and extension, in the flexion state, that is, when the displacement of the four models is at a maximum, the maximum displacement of USS in the four models is the smallest, indicating that USS has better stability.

At present, finite element analysis is more common in orthopaedics, and can explain clinical problems and provide a reference for clinical medical activities. However, due to the complexity of the human body structure, the finite element analysis method has certain limitations. The model is simplified and the material properties differ, which may lead to errors in the experimental results. However, because the finite element method is relatively simple, it can explain some practical problems and plays a very important role in the clinic.
